# Should we perform baseline NGS testing in precursor T lymphoblastic leukaemias: a single centre experience from Eastern India

**DOI:** 10.3332/ecancer.2024.1815

**Published:** 2024-12-06

**Authors:** Prateek Das, Sujeet Kumar, Raghwesh Ranjan, Pradeep Arumugam, Nilesh Dhole, RohitKumar Kori, Anil Yadav, Anil Singh, Vikramjit Kanwar, Neha Singh

**Affiliations:** 1 Hematopathology (Oncopathology), Homi Bhabha Cancer Hospital, Varanasi 221010, India; 2 Homi Bhabha National Institute, Mumbai 400094, India; 3 Department of Medical Oncology (Adult Hematolymphoid Unit), Homi Bhabha Cancer Hospital, Varanasi 221010, India; 4 Department of Pediatric Oncology, Homi Bhabha Cancer Hospital, Varanasi 221010, India; 5 Hematopathology, Homi Bhabha Cancer Hospital, Varanasi 221010, India; 6 Cancer Cytogenetics, Homi Bhabha Cancer Hospital, Varanasi 221010, India

**Keywords:** next generation sequencing, minimal residual disease, precursor T- lymphoblastic leukaemia, cytogenetics, mutations, relapse

## Abstract

**Introduction:**

T-lymphoblastic leukaemia accounts for approximately one-fourth of acute lymphoblastic leukaemia cases. Sequencing approaches have identified >100 genes that can be mutated in T-cell acute lymphoblastic leukaemia (T-ALL). However, the revised WHO 2022 edition of lymphoid neoplasms still does not incorporate molecular signatures into the T-ALL subgrouping unlike B-ALLs and acute myeloid leukemia, which are classified mainly based on molecular landscapes.

**Methods:**

This retrospective observational study included all newly diagnosed patients of T-lymphoblastic leukaemia of all age groups who presented during the period between January 2022 and October 2023 in whom complete baseline diagnostic work-up was available including flow cytometry, fluorescence in situ hybridization and next generation sequencing studies.

**Results:**

There was a lower frequency of karyotypic abnormalities in adult early T progenitor (ETP)-ALLs than in other sub-groups. Non-ETP ALLs showed significant association with NOTCH1 mutations (*p* ≤ 0.00001), followed by JAK3 (*p* = 0.01), FBXW7 (*p* = 0.066) and PHF6 (*p* = 0.09) mutations. There was no difference between adult and pediatric patients, in terms of genomic profiling except in the PHF6 gene. There was no significant difference between NOTCH1-mutated and NOTCH1-wild T-ALL patients as well as NOTCH1-heterodimerization versus NOTCH1-PEST mutated patients in terms of measurable residual disease (MRD), relapse-free survival (RFS) and/or overall survival (OS). 45.1% of all TALL patients harboured ≥3 mutations. However, the complex molecular profile did not correlate significantly with MRD positivity and poor RFS and/or OS rates.

**Conclusion:**

Molecular profiling of TALLs do not significantly impact long-term survival outcomes. In resource-constrained settings, we can get away by not doing comprehensive molecular profiling of TALLs at baseline and restrict the sequencing assay to only those cases that are persistently MRD positive or have relapsed.

## Introduction

T-cell acute lymphoblastic leukaemia (T-ALL) is an uncommon, yet aggressive leukaemia that accounts for approximately one-fourth of acute lymphoblastic leukaemia (ALL) cases. Early studies suggested that Early T cell Precursor (ETP)-ALL is a high-risk disease with a high likelihood of induction failure, chemoresistance, high levels of measurable residual disease (MRD), frequent relapses and inferior clinical outcomes [1, 2]. However, subsequent studies using a risk-adapted approach with more intensified therapy demonstrated conflicting results: some showed no significant differences in event-free survival or overall survival (OS) between ETP-ALL and other T-ALLs, whereas others continued to show poor OS [3–8].

Sequencing approaches focusing on candidate oncogenes, or more recently, genome-wide sequencing, have identified >100 genes that can be mutated in T-ALL. Understanding the molecular aberrations may lead to more targeted therapies and improvement of clinical outcomes [9, 10]. However, the revised WHO classification of lymphoid neoplasms still does not incorporate molecular signatures into the T-ALL subgrouping (ETP versus Non-ETP) unlike B-ALLs and acute myeloid leukemia (AML), which are classified mainly based on molecular landscapes [11, 12]. With this in the background, the primary aim of the study was to study the molecular landscape of our cohort of patients with T-lymphoblastic Leukaemia at diagnosis and their correlation with T-MRD studies and survival outcomes.

## Materials and methods

This retrospective observational study included all newly diagnosed patients of T-lymphoblastic leukaemia of all age groups who presented during the period between January 2022 and October 2023 in whom complete baseline diagnostic work-up was available including flow cytometry, fluorescence *in situ* hybridization (FISH) and next generation sequencing (NGS) studies. Patients with partially-treated TALL from outside as well as relapsed TALLs were excluded from this study. Patients were categorised in accordance with the revised 2022 WHO classification of lymphoid neoplasms. Ethical clearance was obtained for the conduct of the study.

FISH studies were performed on heparinised bone marrow aspirate or peripheral blood of patients using commercially available disease-specific probes (deletion, break-apart and centromeric) according to the manufacturer’s protocol.

For NGS assay, DNA extracted from bone marrow/peripheral blood sample was used as a template. DNA was fragmented which was followed by end repair, A tailing and adapter ligation. Whole genome libraries with patient-specific indices were amplified, followed by hybridization with 1,313 hybrid capture oligonucleotide probes to sequence a 247.74 kb panel of 70 genes. The genes include ABL1 (exons 1–11), ANKRD26 (exons 1–34), ASXL1 (exons 1–13), ASXL2 (exons 1–11), ATM (exons 2–63), ATRX (exons 1–35), BCOR (exons 2–15), BCORL1(exons 2–15), BIRC3 (exons 2–9), BRAF (exons 1–18), BRINP3 (exons 2–7), CALR (exons 1–9), CBL (exons 1–16), CBLB (exons 1–19), CBLC (exons 1–10), CDKN2A (exons 1–3), CEBPA (exon 1), CSF3R (exons 3–18), CUX1 (exons 1–21), DDX41 (exons 1–16), DNMT3A (exons 1–18), ETNK1 (exons 1–8), ETV6 (exons 1–8), EZH2 (exons 2–20), FLT3 (exons 1–24), FBXW7 (exons 1–11), GATA1 (exons 2–6), GATA2 (exons 2–7), GNAS (exons 1–13), HRAS (exons 2–5), IDH1 (exons 1–10), IDH2 (exons 1–9), IKZF1 (exons 2–7), JAK2 (exons 2–25), JAK3 (exons 2–24), KDM6A (exons 1–30), KIT (exons 1–21), KMT2A (exons 2–36), KRAS (exons 2–5), MPL (exons 1–12), MYD88 (exons 1–5), NF1 (exons 1–57), NOTCH1 (exons 1–34), NPM1 (exons 1–12), NRAS (exons 2–5), PDGFRA (exons 2–24), PHF6 (exons 2–10), PIGA (exons 1–6), POT1 (exons 5–18), PPM1D (exons 1–6), PTEN (exons 1–9), PTPN11 (exons 1–15), RAD21 (exons 2–14), RET (exons 1–15,19,20), RUNX1 (exons 2–6), SETBP1 (exons 2–6), SF3B1 (exons 1–20), SH2B3 (exons 1–15), SMC1A (exons 1–26), SMC3 (exons 1–29), SRSF2 (exons 1–2), STAG1 (exons 2–34), STAG2 (exons 2–35), TET2 (exons 3–11), TP53 (exons 1–11), U2AF1 (exons 1–8), WT1 (exons 1–9), XPO1 (exons 2–25), ZBTB7A (exons 2–3) and ZRSR2 (exons 1–11). Sequencing was performed on Illumina MiniSeq using the P2 150-cycle chemistry. Bioinformatics was obtained by Illumina-based solutions. Finally, the variants were annotated and prioritised with population frequency databases (1,000 G (African, Admixed American, East Asian, Finnish, Non-finnish European, South Asian), Exome Aggregation Consortium datasets, NHLBI-ESP (6,500 genomes)) and effect-based databases including ClinVar, Ensembl VEP, Franklin and Varsome as well as the COSMIC databases.

**Supplementary Figure 1.**
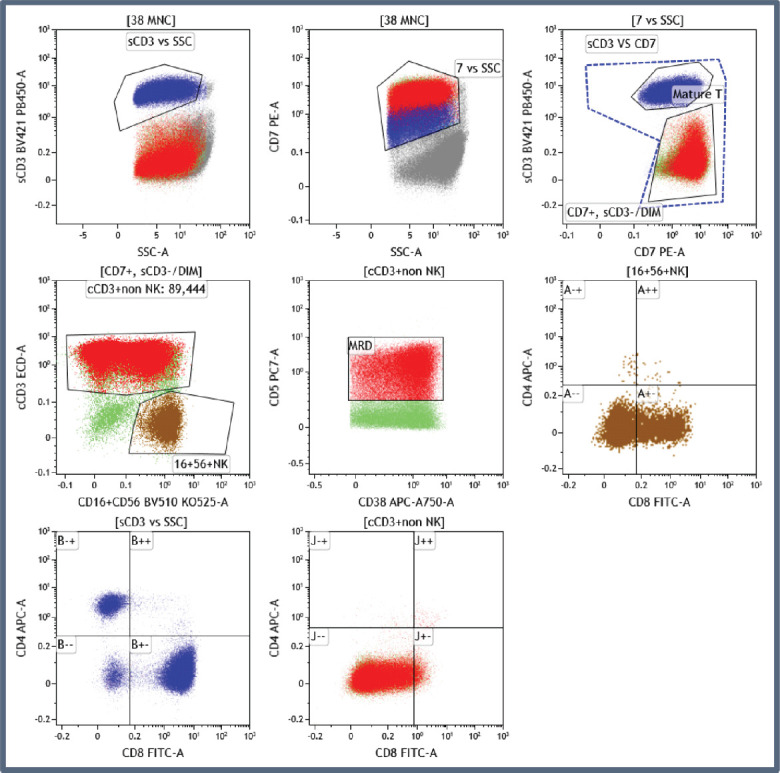
Illustration of the T-MRD positive population marked in red, differentiating them from NK cells, mature T cells and myeloblasts in a non-ETP-ALL patient.

Diagnostic and post-induction MRD immunophenotyping was performed on bone marrow aspirate sample using bulk lysis staining method and further acquired on 3-laser, 13-color Dx FLEX flowcytometer (Beckman Coulter, USA) using cyt expert acquisition software. The 10-color antibody panel for T-MRD studies is shown in Supplementary Table 1. At least 100,000 events were acquired during diagnostic work-up and 1 million events during MRD assessment in each tube. Note that only the first pull bone marrow aspirate was submitted for Measurable residual disease detected by multi-parametric flowcytometry. Immunophenotyping data was analysed with Kaluza (v 2.1) software (Beckmann Coulter, USA), using a pre-defined template-based approach (Supplementary Figure 1).

Patients were treated with Intermediate-risk Berlin Frankfurt Munich (BFM)-95 protocol in adults ≥15 years and High-risk-ICiCLe protocol in pediatric patients <15 years. MRD assessment was done by flow cytometry post-induction (after Induction IA in BFM-95) and post-consolidation (after Induction IIA in BFM-95 protocol). Pediatric patients went for post-consolidation MRD studies only if PI-MRD was positive. The adult patients who were MRD positive after consolidation were counselled for high-risk BFM-95 protocol and stem cell transplant. However, none of the patients opted for bone marrow transplantation due to financial constraints or non-availability of a suitable donor. They were hence continued on BFM protocol, if morphological bone marrow remission was achieved (BM blasts <5% confirmed by flow cytometry), or else they were treated with palliative intent and taken off BFM protocol. Relapse-free survival (RFS) was calculated from the date of complete remission (CR) until the time to relapse or death or last follow-up if in CR.

Data were analysed by using SPSS Software version 23 and presented in median (minimum–maximum), and frequency percentage. The association between categorical variables was tested by the Fisher exact test and chi-square test. All tests were 2-sided and *p* value <0.05 was considered statistically significant. Univariate analysis of the MRD assays and cytogenetic and molecular risk groups was analysed for their impact on RFS using the Kaplan–Meier technique and compared using the log-rank test.

## Results

Out of a total of 71 newly diagnosed T-ALLs fulfilling the inclusion criteria, 48 were adults and 23 were pediatric patients. The frequency of ETP-ALLs was higher among adults (58.3% versus 26.1%) and was associated with severe anemia and neutropenia. Their detailed demographic and baseline hematologic features have been shown in Table 1.

Among the adult T-ALLs, T cell receptor (TCR): A/D rearrangement was the commonest abnormality detected by FISH, unlike the pediatric cohort, in which del9p followed by TLX3 rearrangements were more evident. There was a lower frequency of karyotypic abnormalities in adult ETP-ALLs than in other sub-groups (Supplementary Table 2). As shown in Table 2, ETP-ALLs showed a trend towards higher chances of positive PI-MRD >0.01% (*p* = 0.066) in adult patients and steroid refractoriness (Day +8 blast count) in pediatric patients (*p* = 0.056); however, there was no significant difference between different age groups and WHO-defined subgroups in terms of induction failure rates, RFS or OS.

**Figure 1.**
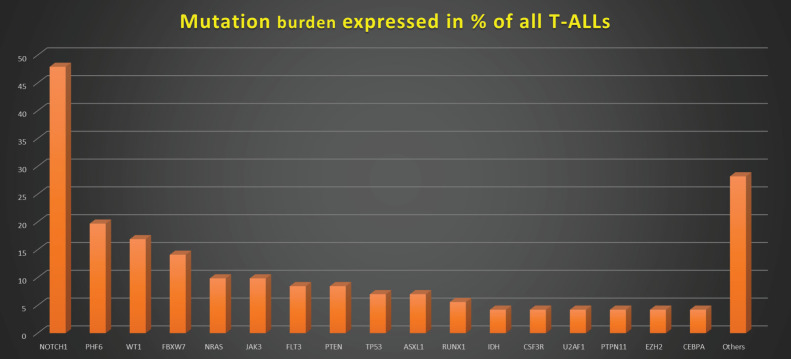
Mutation burden expressed in % of all T-ALLs.

**Figure 2.**
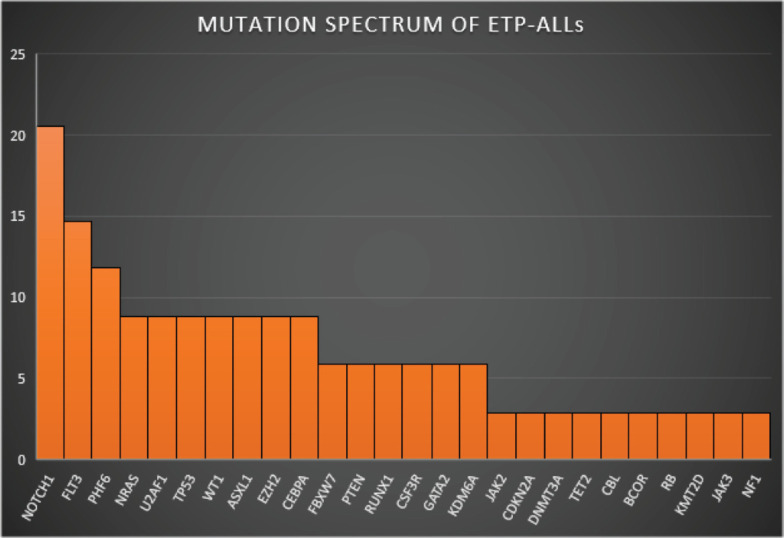
Mutation spectrum of ETP-ALLs.

**Figure 3.**
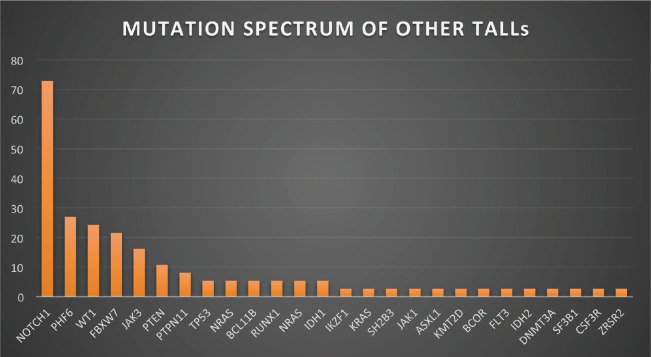
Mutation spectrum of other TALLs.

Figures 1–3 show the diagrammatic representation of the mutational spectrum and frequency of all TALL patients as well as in ETP-ALLs versus Other TALL subgroups, which were not found to be significantly different across all age groups except NOTCH1 mutations as shown in Table 2. Overall, other ALLs showed significant association with NOTCH1 mutations (*p* ≤ 0.00001), followed by JAK3 (*p* = 0.01), FBXW7 (*p* = 0.066) and PHF6 (*p* = 0.09) mutations. On the contrary, ETP-ALLs showed a predilection towards molecular signatures involved in AML leukemogenesis such as FLT3, NRAS, WT1 and TP53. There was no difference between adult and pediatric patients also, in terms of genomic profiling except in PHF6 gene. PHF6 mutations commonly involved exon 8 followed by exons 2, 7 and 9 and were frame-shift insertions or stop-gain mutations in adult patients, unlike the high frequency of mis-sense mutations involving exon 9 in pediatric patients. The VAF% varied between 8.3% and 90.9%.

NOTCH1 mutations occurred at an overall frequency of 47.9% (34/71) in our TALL patients, irrespective of age or subtypes. NOTCH1 gene mutations mainly affected the heterodimerization (HD) domain involving exons 26 and 27 (58.8%; 20/34) and PEST domain involving exon 34 (29.4%; 10/34). 11.8% NOTCH1-mutated patients involved both HD and PEST domains. Mutations were mostly frameshift insertions and/or mis-sense point substitutions. VAF% in NOTCH-mutated patients varied from 5.83% to 51.9%. Two patients showed multiple NOTCH1 mutations. Two patients showed induction failure in each of the three NOTCH-mutated subgroups, i.e., HD, PEST and both. Four patients with NOTCH1-HD mutations and one patient with NOTCH1-PEST mutation had >0.01% MRD-PI positivity, respectively. Four NOTCH1-mutated patients were MRD positive while ten NOTCH1-unmutated patients were MRD positive (*p* value = 0.1). None of the NOTCH mutated patients showed relapse or died. There was no significant difference between NOTCH1-mutated and NOTCH1-wild T-ALL patients as well as NOTCH1-HD versus NOTCH1-PEST mutated patients in terms of MRD, RFS and/or OS.

The molecular spectrum of TALLs is complex which can be elucidated by the fact that 45.1% of all patients harboured ≥3 mutations; however, on univariate analysis, the complex molecular profile did not correlate significantly with MRD positivity and poor RFS and/or OS rates.

## Discussion

We retrospectively analysed the molecular landscape of TALL patients and tried to find out the correlation with MRD and/or treatment outcomes. Our ETP-ALL patients were enriched in myeloid-associated mutations such as *FLT3*, *NRAS*, *WT1, U2AF1* and *TP53*, with relatively reduced frequency of gene mutations typically seen in other subtypes of T-ALL, such as *NOTCH1, FBXW7* and *JAK3*, similar to the findings of Ye *et al* [13]. We found no association between MRD positivity, RFS and/or OS and the mutation status of genes assessed including *NOTCH1*, *PHF6*, FLT3 and *WT1* and also detected no differences in survival between patients with one or two mutations and patients with three or more mutations *similar to the results of their study*. The only difference was in the higher mutation burden in their ETP ALL patients and adverse prognostic impact of TP53 mutation in comparison to our study in which both ETP and Non-ETP patients had equivalent mutational burden (Table 2), and prognostic significance of TP53 mutation could not be ascertained due to paucity of TP53 mutations [13, 14].

85.9% of our patients had at least one mutation, and 45.1% had ≥3 mutations. The genes with higher mutation frequency were *NOTCH1*, PHF6 and WT1, in comparison to the results of a study by Yin *et al* [15] in which 73.3% of patients had at least one mutation, and 36.7% had ≥3 mutations. The genes with higher mutation frequency in their study were *NOTCH1*, *FBXW7* and *DNMT3A* [15].

*NOTCH1* gene consists of extracellular N-terminal epidermal growth factor-like repeats, LIN-12/Notch related repeat domains, HD and C-terminal intracellular domains with PEST motif. Some authors reported that *NOTCH1* mutations correlated with favourable long-term outcomes in paediatric T-ALL [16, 17] while others found no association of NOTCH1 mutation status with treatment outcomes [13]. We did not observe a correlation between *NOTCH1* mutations and survival outcomes. Our findings were in striking contrast to that of Yuan *et al* [18] and Burns *et al* [19] who identified *NOTCH1* gene status and MRD post-induction as independent prognostic factors and favoured inclusion of NOTCH1 mutation status as a risk stratification factor in TALL. The discrepancy may be due to different types of mutations, different ethnic populations and different treatment regimens.

*NOTCH1* mutations in our study were located at either HD domain involving exons 26 and 27 (58.8%) or PEST domain involving exon 34 (29.4%). 11.8% NOTCH1-mutated patients involved both HD and PEST domains. The only Indian study concerning the same agenda by Bhatia *et al* [20] showed NOTCH1 mutations in 52% of their pediatric TALLs, of which 71% were in HD domain and 35% in PEST domain (including one case with mutations in all three domains). They showed that cases with PEST domain NOTCH mutations had poor RFS but the OS was not influenced by NOTCH mutation positivity. We differ on this aspect as RFS and/or OS was not affected by HD or PEST domain of NOTCH mutations in our patients.

*PHF6* gene is an X-linked tumour suppressor involved in the pathogenesis of T-ALL. In T-ALL, there have been mixed reports regarding its prognostic roles; some demonstrated inferior outcomes in patients with mutated *PHF6* [21] whereas others showed no significant differences in outcomes from those with wild-type *PHF6* [22, 23]. We detected *PHF6* mutations in 11.8% of our ETP-ALLs and 27% of Other TALL cases, including nonsense and frameshift mutations in adults and mis-sense mutations in pediatric patients and all occurred in conjunction with other mutations, such as *NOTCH1* and *WT1*. No impact on OS was observed, again likely compounded by the relatively small sample size in the current study [13].

The *WT1* gene is mutated in approximately 10% of TALL cases, and comprised of mainly heterozygous frameshift mutations that cluster in exon 7 and are predicted to lead to a truncated protein Moreover, *WT1* is shown to be among the frequently altered genes in the ETP ALL subgroup. In T-ALL, studies on the role of *WT1* mutations are still limited. In pediatric and adult T-ALL, the presence of *WT1* mutations have not been predictive of poor clinical outcome by Bordin *et al* [24]. In our cohort, WT1 mutations were clustered in exons 7, 8 and 13 and were mostly frameshift indels. The overall frequency of WT1 mutations in TALLs was higher (16.9%) in comparison to their study. Also, the frequency in other TALLs (24.3%) was higher than in ETP-ALLs (8.8%) in our cohort contrary to their findings. However, we also could not establish any impact of WT1 mutations in TALLs on survival outcomes irrespective of age or subtype.

On the basis of the above findings, it may be right to say that molecular profiling of TALLs do not significantly impact long-term survival outcomes, similar to most recently published literature and hence does not find a place in the WHO revised classification of TALLs. That may also be due to the small sample size and short follow-up duration which is the only limitation of this study. In resource-constrained settings, we can get away by not doing comprehensive molecular profiling of TALLs at baseline and restrict the sequencing assay to only those cases that are persistently MRD positive or have relapsed. Also, the intensive treatment regimens nullify the effects of complex molecular landscape to a great extent. It is the largest study on TALL molecular landscape from India and more such studies will pave the way for a clear understanding of TALL biology of Asian patients.

## Conflicts of interest

The authors state that there is no conflict of interest present.

## Funding

NA.

## Consent for publication

It is confirmed that this work is original and has not been published elsewhere, nor is it currently under consideration for publication elsewhere. The manuscript has been read and approved by all the authors.

## Ethical approval

All procedures performed in studies involving human participants were in accordance with the ethical standards of the institutional and/or national research committee and with the 1964 Helsinki Declaration and its later amendments or comparable ethical standards.

## Figures and Tables

**Table 1.** Baseline demographic and hematologic characteristics of T-ALL patients at presentation.

Variables	Adult (*n* = 48)		Paediatric (*n* = 23)	
	ETP-ALL (*n* = 28)	Others (*n* = 20)	ETP-ALL (*n* = 6)	Others (*n* = 17)
Median age (yrs)	25 (16.70)	21 (16–63)	12.5 (3–15)	10 (3–15)
Mean Hb ± SD	7.86 ± 2.14	9.79 ± 2.97	9.5 ± 3.08	9.6 ± 3.03
Median TLC	19 (0.46–375.1)	54 (1.38–485)	65 (19–172)	10.91 (2.24–638)
Median PLT	70 (10–378)	49 (5–534)	87.5 (11–218)	65 (10–241)
Median blasts (%)	67.4	71.5	47	89

**Table 2.** Prognostic factors in T-ALL.

Variables	Adult (*n* = 48)			Paediatric (*n* = 23)		
	ETP-ALL (*n* = 28)	Others (*n* = 20)	*p* value	ETP-ALL (*n* = 6)	Others (*n* = 17)	*p* value
Steroid refractoriness	6 (21.42%)	6 (30%)	0.45	4 (66.7%)	4 (23.5%)	0.056
PI-MRD>0.01%	7 (25%)	1 (5%)	0.066	1 (16.6%)	5 (29.4%)	0.54
Induction failure (≥5 bone marrow blasts confirmed by flow cytometry)	6 (21.42%)	1 (5%)	0.11	1 (16.6%)	3 (17.6%)	0.95
NOTCH1- mutated	5 (17.8%)	17 (85%)	<0.001	2 (33.3%)	10 (58.8%)	0.28
High mutation burden (≥3 mutations)	11 (39.2%)	10 (50%)	0.46	4 (66.6%)	7 (41.2%)	0.28
Relapse	1 (3.57%)	1 (5%)	0.8	1 (16.6%)	1 (5.88%)	0.42
Dead	4 (14.3%)	1 (5%)	0.8	Nil	2(11.7%)	
Median follow up (Range) in months	12 (6–24)	16 (8–28)		20.5 (12–26)	20 (7–28)	

## Supplementary tables and figure

**Supplementary Table 1.** 10- color antibody panels used for bone marrow TALL-MRD assessment.

Fluorochrome	BV510	BV421	FITC	PE	ECD	PC5.5	PC7	APC	APCAF_700	APCAF_750
**Antibody**	CD16+56	CD3	CD8	CD7	CD3 (CYTO)	CD34	CD5	CD4	CD45	CD38
**Clone**	3G8+ NCAM16.2	UCHT-1	A07748	IM1429U	A07748	8G12	A21690	IM2468	A71117	A86049

**Supplementary Table 2.** FISH abnormalities in different T-ALL categories.

FISH abnormalities	Adult (*n* = 48)		Paediatric (*n* = 23)	
	ETP-ALL (*n* = 28)	Others (*n* = 20)	ETP-ALL (*n* = 6)	Others (*n* = 17)
del9p	11 (39.2)	3 (15)	1 (16.6)	6 (35.3)
TCR:A/D	13 (46.4)	8 (40)		3 (17.6)
TCR:B	3 (10.7)	1 (5)		2 (11.7)
TLX3	9 (32.1)	2 (10)		4 (23.5)
TP53 deletion	1 (3.6)		1 (16.6)	
MLL-r	2 (7.1)	2 (10)		
t(9;22)	2 (7.1)			
del(5q)	1 (3,6)			
Negative	17 (60.7)	7 (35)	4 (66.6)	6 (35.3)
Hyperdiploidy	2 (7.1)			
